# A mixed methods observational simulation-based study of interprofessional team communication

**DOI:** 10.1186/1757-7241-21-S2-A1

**Published:** 2013-09-09

**Authors:** Charlotte Paltved, Kurt Nielsen, Peter Musaeus

**Affiliations:** 1 SkejSim Medical Simulation and Skills Training, Aarhus, Denmark; 2 Centre for Medical Education, Aarhus University, Aarhus, Denmark

## Background

Interprofessional team communication has been identified as an important focus for safety in medical emergency care. However, in-depth insight into the complexity of team communication is limited. Video observational studies might fill a gap in terms of understanding the meaning of specific communication interactions and link team performance to patient outcome.

This study had two aims. First, to develop a theory-based evaluation instrument that measures and qualifies team communication. And second, to investigate the quality and content of summaries and re-evaluations evolving step wise and progressively when treating the critically ill patient.

## Methods

The study used mixed methods. The research question sets out to identify which factors most strongly mediate effective and safe team performance.

Team communications were video observed in 29 scenarios. Data analysis employed a grounded theory approach. Communication events and communication failures were recorded and classified into four categories. Furthermore, data supported the building of the SkejSim Team Step Model that captures and conceptualizes the quality of summaries and re-evaluations.

## Results

In the 29 simulations, 1091 communication events and 58 communication failures were recorded and classified. Failure types included “occasion” where timing was suboptimal, “content” where information was inaccurate or missing, “purpose” where issues were not resolved, and “audience”, where a key team member was not present. Two thirds of these failures resulted in visible effects: inefficiency, delay, tension, and procedural error.

Teams were found to differ and these differences could be explained using the five-level model.

## Conclusion

The study found that complex interprofessional team communication does not readily reduce to mere observation and recording of events. An interpretative approach is required to meaningfully account for communication exchanges in context. Despite the complexity of interprofessional team communication, the integration of these two models might provide a significant framework for the construct of efficient team performance. This research has advanced evaluation of team communication, by allowing us to recognize and represent communication by complexity rather than by reductionism and oversimplification. Yet, each aspect is definable and easy to explain and demonstrate to clinicians and thus, holds the promise for simulation-based team training to improve interprofessional team communication.

